# Beneficial Effects of Table Grape Use on Serum Levels of Omega-3 Index and Liver Function: A Randomized Controlled Clinical Trial

**DOI:** 10.3390/biomedicines10092310

**Published:** 2022-09-16

**Authors:** Maria Notarnicola, Valentina De Nunzio, Tamara Lippolis, Valeria Tutino, Anna Maria Cisternino, Palma Aurelia Iacovazzi, Rosa Anna Milella, Marica Gasparro, Roberto Negro, Maurizio Polignano, Maria Gabriella Caruso

**Affiliations:** 1 Laboratory of Nutritional Biochemistry, National Institute of Gastroenterology IRCCS “S. de Bellis” Research Hospital, 70013 Castellana Grotte Bari, Italy; valentina.denunzio@irccsdebellis.it (V.D.N.); tamara.lippolis@irccsdebellis.it (T.L.); 2 Ambulatory of Clinical Nutrition, National Institute of Gastroenterology IRCCS “S. de Bellis” Research Hospital, 70013 Castellana Grotte Bari, Italy; valeria.tutino@irccsdebellis.it (V.T.); annamaria.cisternino@irccsdebellis.it (A.M.C.); gabriella.caruso@irccsdebellis.it (M.G.C.); 3 Laboratory of Clinical Pathology, National Institute of Gastroenterology IRCCS “S. de Bellis” Research Hospital, 70013 Castellana Grotte Bari, Italy; mina.iacovazzi@irccsdebellis.it; 4 Research Centre for Viticulture and Enology, Council for Agricultural Research and Economics, Turi, 70010 Bari, Italy; rosaanna.milella@crea.gov.it (R.A.M.); marica.gasparro@crea.gov.it (M.G.); 5 Personalized Medicine Laboratory, National Institute of Gastroenterology IRCCS “S. de Bellis” Research Hospital, Via Turi 27, Castellana Grotte, 70013 Bari, Italy; roberto.negro@irccsdebellis.it; 6 Clinical Research Unit, National Institute of Gastroenterology IRCCS “S. de Bellis” Research Hospital, Via Turi 27, Castellana Grotte, 70013 Bari, Italy; maurizio.polignano@irccsdebellis.it

**Keywords:** table grape, polyphenols, fatty acids, Omega-3 index, liver disease

## Abstract

This clinical trial was aimed to investigate the effects of fresh table grape intake on the serum levels of the Omega-3 index, defined as the sum of eicosapentaenoic acid (EPA) + docosahexaenoic acid (DHA) levels. Forty consecutive healthy subjects were randomly assigned to the control group, receiving only dietary recommendations, and the grape group receiving a daily dose of 5 g of fresh table grape per kg of body weight, for 21 days. Compared with baseline, the grape treatment produced no significant difference in the serum levels of glucose, liver transaminase, and triglycerides, with the exception of cholesterol value, which was significantly reduced in both control and grape group (180.5 ± 20.32 vs. 196.1 ± 30.0 and 181.4 ± 21.9 vs. 194.3 ± 37.5, respectively). After 4 weeks from the end of grape treatment, the analysis of single fatty acids showed a significant increase in oleic acid content (14.15 ± 1.8 vs. 12.85 ± 1.6, *p* < 0.05) and a significant induction of the Omega-3 index (8.23 ± 1.9 vs. 6.09 ± 1.2, *p* < 0.05), associated with increased serum levels of adiponectin (24.09 ± 1.08 vs. 8.8 ± 0.7, *p* < 0.001). In contrast, the expression of fibroblast growth factor 21 (FGF21), a molecule associated with metabolic syndrome and liver disease, was significantly reduced (37.9 ± 6.8 vs. 107.8 ± 10.1, *p* < 0.001). The data suggest that the intake of fresh grape improves the Omega-3 index in the serum and exerts beneficial effects on liver function through the overexpression of adiponectin and the reduction in FGF21 levels.

## 1. Introduction

Fresh table grape is a rich source of bioactive components, particularly polyphenols, with antioxidant and anti-inflammatory activities [1,2]. A regular consumption of dietary polyphenols is associated with improvements in lipid profile, insulin sensitivity, and with the reduction in the metabolic risk [3,4,5]. The protective role of dietary polyphenols in health has been widely demonstrated [4,5,6,7], as well as their role in modulating gastrointestinal function and inflammation [8,9,10].

Experimental in vitro studies have shown an increase in total polyunsaturated fatty acids (PUFAs) levels in cells treated with Autumn Royal table grape extracts [11,12]. In particular, compared to untreated control cells, the grape extracts caused an increase in essential fatty acids (EFAs), namely linoleic acid (LA) and α-linolenic acid (ALA). In addition, in cell membranes, the grape treatment exerted a significant increase in total PUFAs, associated with changes in their downstream metabolic pathways [11].

Moreover, several clinical and preclinical studies have suggested that polyphenols-rich foods are capable of modulating the metabolism of Omega-3 PUFAs, specifically they increase blood eicosapentaenoic acid (EPA) and docosahexaenoic acid (DHA) levels and modulate eicosanoid metabolism [13,14,15,16], with a significant impact on biomarkers of oxidative stress and inflammation [7,10].

High circulating levels of Omega-3 PUFAs positively affect the serum content of adiponectin [17], an adipocytokine able to increase the insulin sensitivity, influencing glucose uptake, inhibiting gluconeogenesis [18]. Adiponectin has also been demonstrated to inhibit inflammation, reducing the levels of pro-inflammatory cytokines [19,20].

Recently, the evaluation of serum levels of adiponectin and fibroblast growth factor 21 (FGF21) has been considered a valid tool for identifying the presence of metabolic syndrome in children [21]. FGF21 is a hormone involved in the regulation of lipid and glucose metabolism and its expression is regulated by a variety of physiological conditions [21,22,23]. Due to its association with obesity and liver injury, the high levels of circulating FGF21 are often linked to different dysfunctional metabolic processes [24]. About this, higher serum FGF21 levels have been also associated with worse survival in hepatocellular carcinoma (HCC) patients [25].

The composition of circulating fatty acids is considered as a valid biochemical marker for assessing the physiological status of various fatty acids including their possible correlations with the diet [26,27,28]. Close correlations between the serum levels of Omega-3 PUFAs and metabolic disease-related risk have been observed [29]. Moreover, the Omega-3 index, defined as the sum of EPA+ DHA levels, has been demonstrated to be a novel, physiologically relevant risk factor for cardiovascular diseases [30] with a significant clinical utility.

Therefore, given the relationship between dietary components and serum fatty acids profile, and considering that the effects of table grapes on lipidomic profile needs to be confirmed by clinical studies, the main objective of the study was to investigate, in humans, the effects of dietary intake of fresh table grape on the metabolism of PUFAs, particularly the Omega-3 index.

The aim of this study was also to investigate the impact of dietary grape supplementation on some candidate markers of early metabolic disorders, such as adiponectin and FGF21.

The clinical data that will be obtained from this study could also be useful for identifying patients with metabolic deficits or chronic metabolic diseases who could benefit from a diet enriched with grapes.

## 2. Materials and Methods

### 2.1. Patients

**Figure 1:**
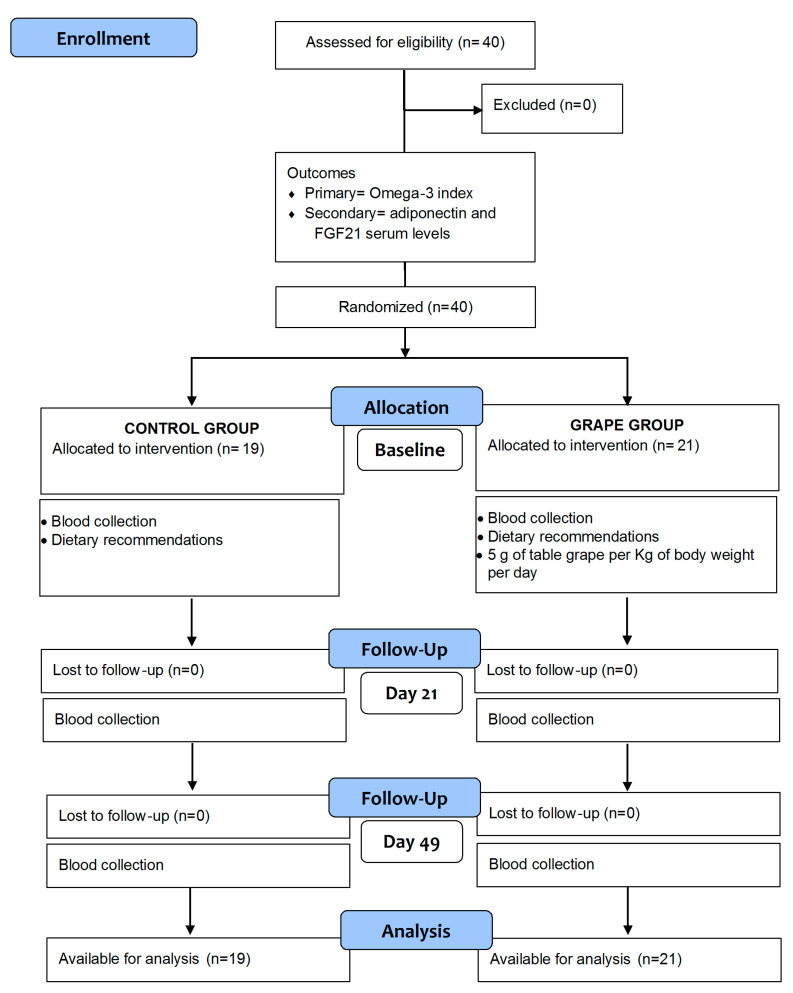
Flowchart of study design (Baseline, T0; Day 21, T1; Day 49, T2).

Forty consecutive healthy subjects, mean age 45.36 ± 10.1 (13 males and 27 females) were recruited on a voluntary basis from the Ambulatory of Nutrition of our Institute from September to December 2019. The clinical trial, registered on http://www.clinicaltrials.gov (6 August 2019) (reg. number: NCT04053569), was approved by the local Scientific Committee and the Institutional Ethics Committee of Istituto Tumori Giovanni Paolo II, Bari, Italy, Prot. N. 79/EC. Written informed consent was obtained from all the subjects for blood testing and clinical data collection. The study was conducted in accordance with the Helsinki Declaration and the participants were randomly assigned to two groups of the study (control and grape group), as previously described [8]. Briefly, the grape group consisting of healthy subjects was invited to daily consumption of 5 g of fresh table grape per kg of body weight for 21 days (T1). Table grape used in this study was the black seedless grape Autumn Royal, an experimental variety whose characteristics have been previously described [8,11]. All enrolled subjects received dietary recommendations, as limitation of alcohol, caffeine, and polyphenol-rich foods. The participants assigned to the control group were asked to abstain from eating grapes. After 4 weeks from the end of grape treatment, all subjects, including the controls, were asked to undergo a blood draw for fatty acids evaluation and biochemical analyses (T2). The clinical trial flowchart is shown in Figure 1 and the adherence to the grape consumption was estimated by an adherence score described in Table S1.

### 2.2. Blood Samples

Blood samples were collected in the morning after 12 h of fasting in vacutainer tubes containing silica gel at T0 (baseline), T1 (day 21), and T2 (day 49). The samples were then centrifuged at 2000× *g* for 10 min at 4 °C to obtain serum and stored at −80 °C until use.

### 2.3. Serum Fatty Acids Extraction

Serum fatty acid extraction was performed using the Fatty Acid Extraction Kit, Low standard (Sigma-Aldrich, St. Louis, MO, USA) according to the manufacturer’s instructions. Briefly, serum samples were treated with 3 mL of Extraction Solvent and 0.5 mL of Aqueous Buffer, vortexed, and placed inside a syringe to elute the lipids. The eluted lipids were dried and esterified with 1 mL of Boron trifluoride-methanol solution and 0.3 mL of hexane. After a period of incubation (1 h at 95 °C), 1 mL of hexane and 1 mL of distilled water were added to the extracted mixture, which was vortexed and centrifuged at 500× *g* for 5 min. The top of the hexane layer was transferred in a new tube and dried. The esterified lipids were reconstituted with 100 µL of hexane and analyzed.

### 2.4. Serum Fatty Acids Analysis and Quantification

Fatty acid methyl esters extracted were analyzed by a gas chromatography equipment with auto-sampler, a split/splitless injector, FID detector, and a hydrogen gas generator (Thermo Fisher Scientific, Milan, Italy). The analysis was carried out on a BPX 70 capillary column (SGE Analytical Science, P/N SGE054623, 60 m × 0.25 mm ID—BPX70 0.25 µM, SGE Europe Ltd., Milton Keynes, UK). Hydrogen was used as carrier gas, 3.0 mL min^−1^, constant flow mode; the amount injected was 1 µL in splitless mode (split flow 50 mL min^−1^, splitless time 1 min). The temperature of the injector and the FID detector were 250 °C and 270 °C, respectively. The initial temperature of the oven was 40 °C, then it increased to 170 °C at 10 °C min^−1^ for 5 min, then to 200 °C at 4 °C min^−1^ for another 5 min, and finally the temperature increased to 255 °C at 50 °C min^−1^ and held for 4.5 min. The identification of each peak was obtained by comparing the retention times with those of a mixture of standards (Supelco 37-Component FAME Mix, Sigma-Aldrich, Milan, Italy). Data were expressed as percentage of each fatty acid calculated on the total amount of fatty acids.

### 2.5. Adiponectin and Fibroblast Growth Factor 21 (FGF21) Assay

Serum Adiponectin and FGF21 levels were evaluated in duplicate using commercially available sandwich enzyme-linked immunosorbent assay kits (Human ADP Adiponectin ELISA kit and Human FGF21 ELISA Kit, respectively, by MyBioSource, San Diego, CA, USA).

## 3. Results

### 3.1. Effect of Table Grape on Serum Biochemical Parameters

Table 1 shows the clinical and metabolic features of the participants who completed the study. Compared to baseline, 21 days of grape treatment produced no significant difference in serum levels of glucose, AST, ALT, and triglycerides. As previously reported, only the cholesterol levels were significantly reduced at T1 compared to T0, in both experimental groups (180.5 ± 20.32 vs. 196.1 ± 30.0 and 181.4 ± 21.9 vs. 194.3 ± 37.5, respectively). Interestingly, at T2, the cholesterol values restored to the levels at the baseline. Moreover, no adverse event was observed after the grape use.

### 3.2. Effect of Table Grape on Serum Fatty Acids Profile

Based on previous preliminary data, the study of fatty acids content in the serum was conducted at T0 and T2 (4 weeks after the end of grape treatment), considered an appropriate time to observe possible changes in fatty acids concentrations.

**Figure 2:**
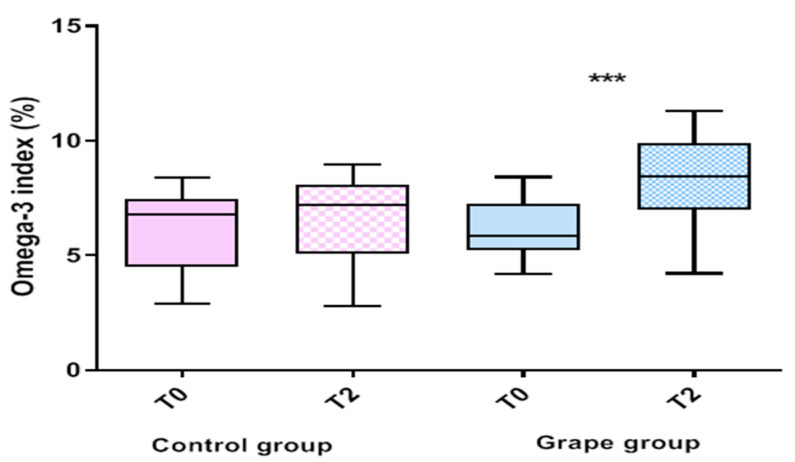
Omega-3 index levels in the serum from control and grape group at baseline (T0) and after 4 weeks from the end of grape treatment (T2). Data expressed as the mean percentage ± standard deviation. *p*-Value was determined by Paired *t*-test; *** *p* < 0.001.

Table 2 shows the serum compositions of single fatty acids detected in two experimental subject groups. In the grape group, a significant effect of table grape intake on EPA and DHA levels was observed and the Omega-3 index increased in a statistically significant manner (6.095 ± 1.27 vs. 8.23 ± 1.89, *p <* 0.001, Paired *t*-test) when compared to the values detected at baseline (Figure 2).

Table grape treatment also induced the serum oleic acid content (14.15 ± 1.8 vs. 12.85 ± 1.6, *p* < 0.05, Paired *t*-test), a monounsaturated fatty acid described to have anti-inflammatory activity. No significant differences in fatty acid levels were observed between baseline and T2 in the control group.

### 3.3. Impact of Table Grape on Serum Adiponectin and FGF21 Levels

**Figure 3:**
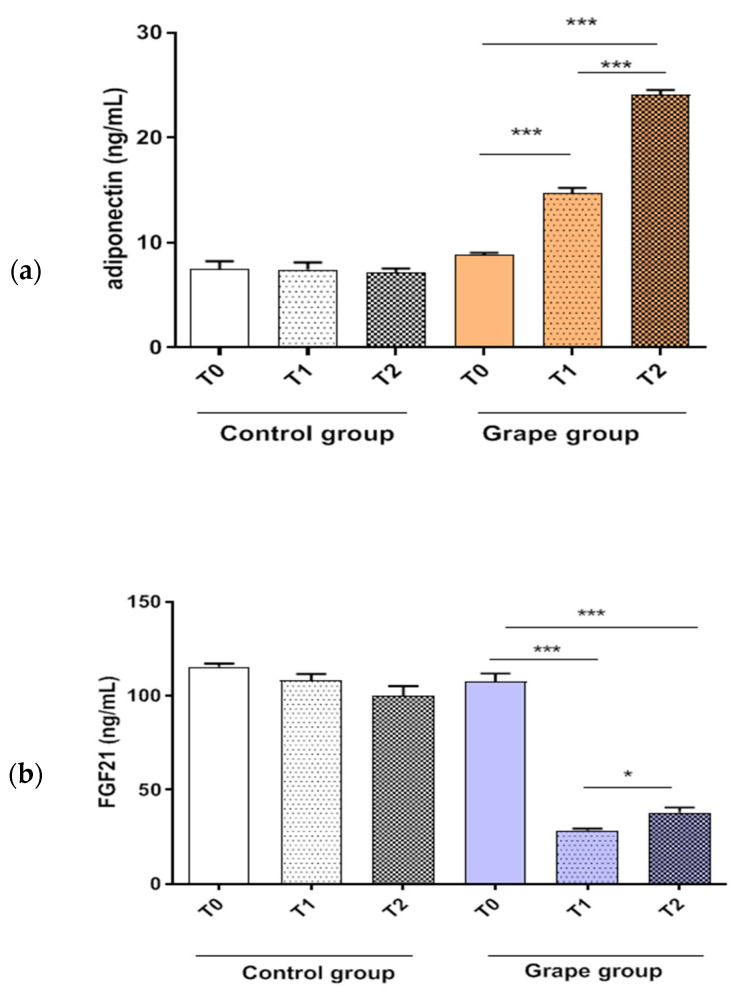
Serum levels of adiponectin (**panel a**) and FGF21 (**panel b**) from control and grape group at T0, T1, and T2. Data expressed as mean ± standard deviation. *p*-Value was determined by Paired *t*-test; * *p* < 0.05; *** *p* < 0.001.

The effect of table grape intake on the serum levels of adiponectin and FGF21 are shown in Figure 3. The grape group presented a significant increase in adiponectin levels compared to baseline. This increase was time-depending, so that the effects were more evident at T2.

Opposite behavior was observed for the expression of FGF21; a statistically significant reduction in FGF21 levels was detected in the serum of subjects treated with the grape-rich diet. The grape effect was slightly more evident at T1 than T2.

Compared to baseline, no difference in serum adiponectin and FGF21 concentrations was observed in the control group, both T1 and T2.

## 4. Discussion

The Mediterranean Diet is characterized by an adequately balanced combination of fruit and vegetables, fish, and cereals rich in polyphenols, fiber, and polyunsaturated fats, contributing to maintaining a healthy status. This dietary pattern is essential as a preventive measure against the onset of cancer and other chronic diseases and to reduce healthcare costs. Consequently, it is necessary to continue investigating the molecular mechanisms whereby the Mediterranean Diet exerts its protective effects.

Polyphenols, secondary plant metabolites, protect and reduce inflammation by different pathways, through mechanisms of down-regulation, balance, and up-regulation, preventing obesity, cancer, and age-related diseases, in which inflammation has an important pathological role [31].

Table grapes, in particular, are typical fruits of the Mediterranean tradition, characterized by a high content of polyphenols. On the effects of grape polyphenols on human health, recently, we carried out some studies both in vivo and in vitro to clarify the molecular mechanisms involved. The Autumn Royal grape had already shown in vitro a great ability to affect cell membrane PUFAs profile, as well as cell morphology and migration [11,12]. In human colorectal cancer cell lines, the treatment with Autumn Royal grape variety exerted a significant increase in total PUFAs and, notably, a significant reduction in the arachidonic acid content. These effects are probably due to its high content in flavonoid compounds, known to be anti-inflammatory and antioxidant agents in the cells.

The Autumn Royal black table grape has been also demonstrated to influence the expression of circulating coding and non-coding RNA sequences, namely microRNAs, involved in the modulation of gastrointestinal cancer-related pathways [8]. The subjects treated with Autumn Royal black grape showed a significant down-regulation of 18 miRNAs involved in pathways related to cancer. The down-regulation of these circulating miRNAs linked to cell proliferation and inflammatory processes demonstrated that dietary components can modulate gene expression, showing specific functional, preventive, and therapeutic effects. Although the effects of these compounds depend upon the amount consumed, their bioavailability and potential interactions with other nutrients, a lot of experimental evidences have confirmed the ability of diet-derived polyphenols to modulate the metabolism of polyunsaturated fatty acids (PUFAs), both Omega-3 and Omega-6 PUFAs [7,10].

In the present study, we demonstrate a significant up-regulation of the Omega-3 index in the healthy subjects, after 4 weeks from the end of grape treatment. Based on previous preliminary data, we considered it an appropriate time to evaluate possible changes in serum fatty acids content.

The mechanisms by which the daily use of grape increases serum EPA and DHA levels are few studied, even if preclinical studies have showed that a diet rich in flavonoids may induce the synthesis of EPA and DHA through the activation of their precursor α-linolenic acid [7]. It has been also demonstrated that flavonoid and not flavonoid compounds of Autumn Royal grape skin extracts modulate membrane PUFAs content in human colon cancer cell lines [11]. These findings observed in vitro have been confirmed in humans, showing the ability of the Autumn Royal grape variety to interfere with the metabolism of PUFAs.

A previous nutrigenomic study [32] has demonstrated that the fresh table grape intake exerted an up-regulation of the fatty acid desaturase 1 (FADS1) enzyme, also known as delta-5 desaturase. This protein is one of the rate-limiting enzymes in the PUFA desaturation pathway, involved in catalyzing the conversion of dihomo-γ-linolenic acid (DGLA) to arachidonic acid (AA), EPA, and DHA. Notably, the dysregulation in FADS1 influences hepatic lipid homeostasis by modulating the PPARα-FGF21 axis [33] and a decreased hepatic FADS1 expression, associated with low levels of long-chain PUFAs, have been detected in subjects with nonalcoholic fatty liver disease (NAFLD).

The significant induction of the Omega-3 index detected in the grape group was associated with higher serum levels of adiponectin, a molecule known to have a key regulatory role in promoting fatty acids oxidation in the liver [34,35]. It has been reported that adiponectin release increases in response to EPA and DHA supplementation [20].

Moreover, experimental evidences have also suggested the ability of adiponectin to inhibit inflammation, modulating the expression of NF-kB and reducing the expression of TNF-α, IL-6, and IL-8 [36].

The anti-inflammatory activity of the grape-rich diet, was reported in Ammollo et al., 2017 [37] with the decreased release of IL-1β in PBMCs in response to in vitro lipopolysaccharide stimulation. Moreover, the array analysis [32] showed the down-regulation of the IL-1β gene and other genes involved in the inflammation, such as some chemokines and their receptors, as well as a down-regulation of the pathways involved in the cellular inflammatory response, such as TNF, NLR, and JAK/STAT. Therefore, high levels of adiponectin observed in the subjects after grape treatment confirm data showing that fruits polyphenols, as well as their metabolites have various beneficial effects on human health.

The reduction in FGF21 is also in line with well-known anti-inflammatory functions of both EPA and DHA, since increased FGF21 levels have been associated with the presence of inflammatory chronic diseases, including metabolic syndrome [20,34]. Furthermore, FGF21 has been found to promote the metabolic events leading to type 2 diabetes mellitus, NAFLD, and obesity [38,39]. Overall, the physiological role of FGF21 is to act on white adipose tissue lipolysis, increase insulin-dependent glucose uptake, and revert insulin resistance [40,41]. In this context, some studies suggest that the FGF21/adiponectin ratio may be a sensitive marker for the evaluation of liver steatosis [21,42].

Based on the data from this study, we can suggest that grape intake could also be beneficial for individuals with impaired liver or glucose metabolism. In this regard, no alteration of the biochemical parameters (as glycaemia, AST, ALT, etc.) was detected after grape consumption suggesting that daily grape intake exerts beneficial effects in maintaining metabolic homeostasis.

However, the study has some limitations due to the need to increase the sample size and to investigate in the serum the bioaccessibility and bioavailability of the bioactive compounds present in the grape. The strength of the study is the evidence which the use of fresh grapes can be considered as a preventive option to reduce the risk or delay the onset of multiple chronic pathological conditions.

## 5. Conclusions

The remarkable impact of the Autumn Royal grape on the stimulation of EPA and DHA synthesis and, consequently, on the increase in the Omega-3 index in the serum, translates into beneficial effects on liver function through the overexpression of adiponectin and the reduction in FGF21 levels.

The results of this study are of interest since they also provide some indications on which molecules to use for future nutrigenomics studies aimed to elucidate how the deficiency of several macronutrients results in significant metabolic disorders.

## Figures and Tables

**Table 1:** Clinical and Biochemical Characteristics of Participants at T0 (Baseline), T1 (Day 21), and T2 (Day 49).

	Control Group (*n* = 19)	Grape Group (*n* = 21)
Sex		
Male	4	9
Female	15	12
Age	44.1 ± 10.14	47.4 ± 9.5
Glucose (mg/dL)		
*T0*	82.53 ± 6.05	79.33 ± 6.08
*T1*	84.05 ± 7.7	79.76 ± 5.43
*T2*	80.00 ± 9.81	77.41 ± 10.67
AST (U/L)		
*T0*	18.89 ± 4.34	18.67 ± 3.54
*T1*	19.9 ± 7.34	18.48 ± 3.65
*T2*	19.07 ± 4.33	19.71 ± 5.64
ALT (U/L)		
*T0*	22.47 ± 10.6	18.62 ± 7.26
*T1*	23.58 ± 12.0	19.9 ± 6.24
*T2*	19.93 ± 7.96	18.53 ± 7.19
γ-GT (U/L)		
*T0*	19.32 ± 8.39	17.20 ± 9.30
*T1*	15.80 ± 7.40	16.20 ± 6.50
*T2*	15.47 ± 7.68	14.65 ± 6.65
Cholesterol (mg/dL)		
*T0*	196.1 ± 30.0	194.3 ± 37.5
*T1*	180.5 ± 20.32 *	181.4 ± 21.9 *
*T2*	188.9 ± 33.96	198.8 ± 41.85
HDL-C (mg/dL)		
*T0*	51.20 ± 12.80	54.62 ± 7.99
*T1*	49.70 ± 10.60	51.77 ± 4.24
*T2*	51.33 ± 11.60	54.88 ± 13.13
LDL-C (mg/dL)		
*T0*	120.1 ± 21.6	116.1 ± 23.24
*T1*	118.3 ± 22.5	118.5 ± 21.5
*T2*	123.1 ± 27.33	120.7 ± 25.24
Triglycerides (mg/dL)		
*T0*	87.50 ± 41.53	69.33 ± 37.34
*T1*	76.60 ± 29.03	67.35 ± 25.49
*T2*	72.53 ± 45.29	71.06 ± 39.53
Protein C reactive (mg/dL)		
*T0*	0.14 ± 0.26	0.14 ± 0.26
*T1*	0.13 ± 0.36	0.18 ± 0.16
*T2*	*0.24 ± 0.67*	*0.10 ± 0.26*

Abbreviations: AST, aspartate transaminase; ALT, alanine aminotransferase; γ-GT, γ-glutamyl-transpeptidase; HDL-C, high-density lipoprotein cholesterol; LDL-C, low-density lipoprotein cholesterol. All values are expressed as mean ± Standard Deviation. * *p* < 0.05, Paired *t*-test.

**Table 2:** Fatty Acids Content in the Serum from Control and Grape Group at Baseline (T0) and after 4 Weeks from the End of Treatment (T2).

Fatty Acid (%)	Control Group		Grape Group	
	T0	T2	T0	T2
Palmitic acid	18.30 ± 2.80	19.98 ± 3.92	18.44 ± 1.91	18.27 ± 2.99
Stearic acid	14.33 ± 1.98	15.51 ± 2.42	14.27 ± 1.83	14.29 ± 2.12
Oleic acid	13.04 ± 1.87	14.38 ± 1.48	12.85 ± 1.60	14.15 ± 1.86 *
Vaccenic acid	0.89 ± 0.23	0.99 ± 0.21	0.83 ± 0.24	0.90 ± 0.35
Linoleic acid	10.61 ± 1.39	10.93 ± 1.21	11.00 ± 1.58	11.45 ± 1.66
γ-linoleic acid (GLA)	0.69 ± 1.74	0.59 ± 1.29	0.34 ± 0.85	0.37 ± 1.45
α-linolenic acid (ALA)	0.11 ± 0.07	0.13 ± 0.14	0.12 ± 0.11	0.16 ± 0.30
Arachidonic acid (AA)	18.25 ± 3.96	19.19 ± 3.43	17.72 ± 1.98	18.84 ± 3.43
Eicosapentaenoic acid (EPA)	1.65 ± 1.09	2.07 ± 1.15	2.02 ± 0.98	2.94 ± 1.18 *
Docosahexaenoic acid (DHA)	4.37 ± 1.53	4.60 ± 2.01	4.07 ± 1.19	5.29 ± 1.45 *
Saturated fatty acids	40.62 ± 3.91	41.67 ± 4.27	41.83 ± 2.82	41.65 ± 5.22
Monounsaturated fatty acids	17.65 ± 1.70	18.01 ± 1.41	17.26 ± 1.29	17.53 ± 1.64
Polyunsaturated fatty acids	38.67 ± 4.88	37.75 ± 4.35	37.76 ± 3.05	38.81 ± 5.71

Data expressed as the mean percentage ± standard deviation (SD); * *p* < 0.05, Paired *t*-test.

## Data Availability

Data is contained within the article.

## Supplementary Materials

The following supporting information can be downloaded at: https://www.mdpi.com/2227-9059/10/9/2310/s1, Table S1: Adherence questionnaire.
