# Supplementary material for: Beneficial Effects of Table Grape Use on Serum Levels of Omega-3 Index and Liver Function: A Randomized Controlled Clinical Trial
Source: Biomedicines. 2022 Sep 16;10(9):2310. doi: 10.3390/biomedicines10092310 (PMC9496466; doi:10.3390/biomedicines10092310)
Supplement: Supplementary file 1 [file biomedicines-10-02310-s001.zip › biomedicines-1872829-supplementary.pdf]

Table S1: Adherence questionnaire

Answers	Always<br>*Score: 1*	Often<br>*Score: 2*	Sometimes<br>*Score: 3*	Rarely<br>*Score: 4*	Never<br>*Score: 5*
I replaced the grapes received with other varieties					
I forgot to eat the received portion of grapes					
I stopped eating the received grapes for a period of time					

The maximum possible score is 15 points. Adherence to the study design is confirmed by a score ≥ 12 (≥80%).
